# Combining Gold Nanoparticles with Other Radiosensitizing Agents for Unlocking the Full Potential of Cancer Radiotherapy

**DOI:** 10.3390/pharmaceutics13040442

**Published:** 2021-03-25

**Authors:** Abdulaziz Alhussan, Ece Pinar Demirci Bozdoğan, Devika B. Chithrani

**Affiliations:** 1 Department of Physics and Astronomy, University of Victoria, Victoria, BC V8P 5C2, Canada; alhussan@uvic.ca (A.A.); ebozodogan@uvic.ca (E.P.D.B.); 2 Division of Medical Sciences, University of Victoria, Victoria, BC V89 5C2, Canada; 3 Center for Advanced Materials and Related Technologies (CAMTEC), University of Victoria, Victoria, BC V89 5C2, Canada; 4 Department of Computer Science, Mathematics, Physics and Statistics, Okanagan Campus, University of British Columbia, Kelowna, BC VIV 1V7, Canada; 5 Division of Medical Sciences, BC Cancer Research Institute, Victoria, BC V8R 6V5, Canada; 6 Center for Biomedical Research, University of Victoria, Victoria, BC V89 5C2, Canada

**Keywords:** radiotherapy, gold nanoparticles, cisplatin, docetaxel

## Abstract

About half of cancer patients (50%) receive radiotherapy (RT) for the treatment of local tumors. However, one of the main obstacles in RT is the close proximity of adjacent organs at risk, resulting in treatment doses being limited by significant tissue toxicity, hence preventing the necessary dose escalation that would guarantee local control. Effective local cancer therapy is needed to avoid progression of tumors and to decrease the development of systemic metastases which may further increase the possibility of resection. In an effort to do so, radiosensitizing agents are introduced to further increase damage to the tumor while minimizing normal tissue toxicity. Cisplatin and docetaxel (DTX) are currently being used as radiation dose enhancers in RT. Recent research shows the potential of gold nanoparticles (GNPs) as a radiosensitizing agent. GNPs are biocompatible and have been tested in phase I clinical trials. The focus will be on exploring the effects of adding other radiosensitizing agents such as DTX and cisplatin to the GNP-RT platform. Therefore, a combined use of local radiosensitizing agents, such as GNPs, with currently available radiosensitizing drugs could make a significant impact in future RT. The ultimate goal is to develop treatments that have limited or nonexistent side effects to improve the quality of life of all cancer patients.

## 1. Introduction

The Canadian Cancer Society estimated that nearly 50% of Canadians will be diagnosed with cancer at some point during their lifetime and 25% of Canadians will die from it. The estimated numbers of new cancer cases and deaths due to cancer in Canada in 2020 was approximately 225,800 and 83,300, respectively. Even though an increasing number of Canadians are surviving at least five years past their cancer diagnosis, cancer continues to be the leading cause of death in Canada (~30%). Radiotherapy (RT) is one of the most widely used cancer treatment modalities ever since X-ray photons were discovered by Wilhelm Röntgen at the end of the 19th century, with about half of cancer patients receiving RT. Based on recent statistics, RT has cured seven times as many patients as chemotherapy [1]. Despite that, we are at the maximum threshold of the RT dose that can be safely given to patients, creating a clear need for novel methods to enhance the effects of RT to further improve the survival rates while reducing the side effects. Therefore, improvements in RT methods using radiosensitizing agents could have a very big impact in the treatment of cancer in the near future.

The goal of curative treatment is to maximize tumor control. It is clear and logical that as radiation dose increases, tumor response also increases. However, because tumors are not perfectly shaped, but are irregular masses seated within the patient surrounded by healthy organs and tissues, the surrounding normal tissue will also, inevitably, receive undesirable doses during any radiation treatment. In the clinic, physicians set the upper limit of tolerance for normal tissue toxicity with one major factor considered being the patient’s quality of life. This limitation in the radiation dose puts a maximum threshold of feasible tumor response. One method to improve the therapeutic index in RT without causing much toxicity to normal cells is the introduction of radiosensitizing agents, specifically, targeted radiosensitizing agents. In this article, we will focus on three different radiosensitizing agents: gold nanoparticles (GNPs), cisplatin, and docetaxel (DTX). Both cisplatin and DTX are chemotherapeutics drugs used in the clinic. GNPs are not currently used in the clinic, but their biocompatibility has been tested in a phase I clinical trial. The future prospects of RT with GNPs in combination with cisplatin or DTX will also be discussed.

## 2. Targeting Strategies for Cancer Treatment

Targeting strategies vary depending on the location and stage of the cancer. For example, geometrically targeted therapies rely on macroscopic properties of cancerous tissue, generally the location or geometry. These treatments include surgical intervention and RT. They can be targeted locally to spare normal tissue from damage, e.g., by not cutting normal tissue, but are unable to treat metastases that are undetectable or outside of the targeted region [2]. Chemotherapy is a functionally targeted treatment which affects a property or function inherent to the cancer cells that is either different from normal cells or expressed to a different degree. Chemotherapy is needed to control the tumor cell population when the tumor is metastasized. Targeting cell division is effective with cancer cells since they generally divide much faster than healthy cells. Chemotherapeutic drugs are usually administered systemically (intravenously or orally). This type of treatment has the advantage of being able to affect tumors throughout the patient including undetectable metastases, but often cause toxicity in normal organs and rapidly-dividing healthy tissues [3].

### 2.1. Measuring Success: The Therapeutic Ratio

**Figure 1:**
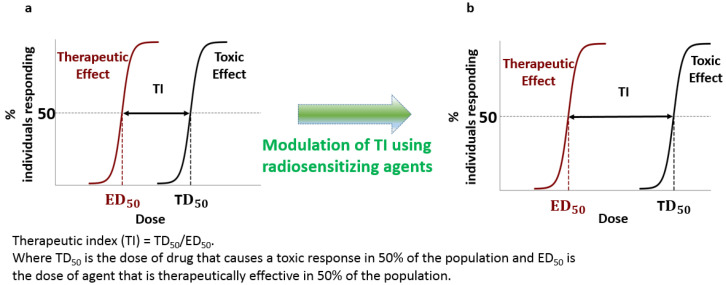
Modulation of therapeutic index (TI) using radiosensitizing agents. (**a**) Variation of the local tumour control probability (red) and normal tissue complication (black) for single treatment option. (**b**) Combination of therapeutic agents could improve the therapeutic window.

The ideal goal of cancer treatment is 100% tumor control, i.e., to halt all division of cancerous tissues by cell death or sufficient damage. Unfortunately, due to the difficulties encountered in targeted cancer therapies, this is often not practically possible as the collateral damage to the patient would cause death or unacceptable side-effects. The therapeutic index (TI) is a tool used to assess the balance between tumor control and patient quality of life. It is a comparison of the amount of a therapeutic agent that causes the therapeutic effect to the amount that causes toxicity (see Figure 1a) [2,4]. In other words, the therapeutic index is a measurement of the contrast a treatment is able to “see” between normal and cancerous cells. Physicians set the upper tolerance for complications and it would limit the achievable tumor response. Therefore, it is critical that we explore other possibilities that increase the therapeutic index while minimizing normal tissue toxicity. Current research strategies to optimize TI is the focus of this article. This should allow us to enhance the therapeutic window (see Figure 1b).

### 2.2. Combining Strategies for Improved Outcomes

One common strategy in chemotherapy is to combine anticancer drugs with non-overlapping mechanisms and profiles of side effects. This would cause maximum damage to the cancer due to the combined effects of the drugs while keeping the side effects more “spread out” over different organs and tissues. This is also a strategy to cope with drug resistance evolving within the tumor [3]. Another strategy is to combine RT and chemotherapy treatments. For example, the chemotherapeutic drug cisplatin is currently added as a radiosensitizer to enhance the radiation dose [5]. DTX is another chemotherapeutic drug which can act as a radiosensitizer. However, it is not heavily used with radiation due to associated side effects. These types of radiosensitizers make cancer cells more vulnerable to radiation, sometimes in addition to other therapeutic properties. Radiosensitization mechanisms vary from directly amplifying the radiation dose or creating additional reactive oxygen species to making the cell ignore DNA damage [6]. These combined therapeutic approaches improve the therapeutic ratio within the irradiated tumor volume [2]. In this article, we will discuss how GNPs can be combined with these radiosensitizing drugs to further improve the TI in RT (see Figure 1b). Currently, we are at the maximum threshold of RT doses given to patients that would cause minimal side effects, any further increase in current radiation doses would very likely cause serious damage to healthy tissues; thus, there is a need for such novel modalities to improve patients’ survival rates.

## 3. Cellular Uptake and Transport of GNPs

**Figure 2:**
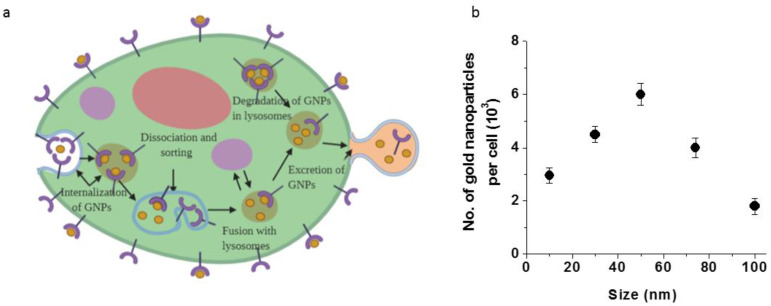
Cellular uptake of GNPs. (**a**) Endo-lyso pathway of GNPs; GNPs are internalized via endocytosis, get processed, and then get excreted out of the cell [7]. (**b**) Size-dependent uptake of GNPs. Reproduced with permission from [13], American Chemical Society, 2006.

In order to produce radiation dose enhancement, GNPs need to accumulate in tumor cells. GNPs enter cells mostly via receptor-mediated endocytosis (RME) and it occurs through interactions between the proteins on the surface of the nanoparticle and receptors on the cell membrane (see Figure 2a). Cell surface receptors bind to molecules on the surface of nanoparticles (NPs) causing membrane wrapping of the NP with a corresponding increase in elastic energy [7]. The receptor-ligand binding immobilizes receptors causing configurational entropy to be reduced. More receptors diffuse to the wrapping site, driven by the local reduction in free energy, allowing the membrane to wrap completely around the particle [8]. Receptor-mediated endocytosis is an energy-dependent process. The path of the NPs within the cell is explained in Figure 2a. Once GNPs are bound to the receptors on the surface of the cell, membrane invagination occurs followed by the trapping of GNPs in endosomal vesicles [9]. These endosomal vesicles or endosomes are categorized sequentially as early endosomes that appear just beneath the plasma membrane, and late endosomes that appear closer to the Golgi apparatus and the nucleus [10]. The interior of endosomes is acidic (~pH 6) due to the H^+^-ATPase in the endosomal membrane and many internalized receptor proteins change their conformation and release their ligand in this acidic environment. Some molecules and receptors in the endosomes are recycled back to the same plasma membrane they came from (recycling) through transport vesicles, some proceed to a different domain of the plasma membrane (transcytosis), and others progress to lysosomes for degradation. GNPs are eventually transported to lysosomes before being excreted from the cell [11].

Competition between membrane wrapping and receptor diffusion dynamics results in a size-dependent uptake of NPs. Studies of different-sized colloidal GNPs showed that the maximum uptake occurred when NPs have 50 nm diameter as shown in Figure 2b. GNPs of this size are able to more efficiently enter cells via receptor-mediated endocytosis [12]. Chithrani et al. hypothesized that the size-dependent uptake of GNPs is mediated by nonspecific and instantaneous adsorption of serum proteins on the surface of GNPs [13]. This group also found that the uptake of transferrin-coated GNPs was three times less than the uptake of unmodified (citrate-stabilized) GNPs and suggested that this is because a diverse set of serum proteins adsorb onto the surface of unmodified (citrate stabilized) GNPs while allowing the GNPs to enter the cells through various corresponding pathways [12,13].

For in vivo applications, it is important to functionalize these NPs with polyethylene glycol (PEG) which increases the blood circulation time of NPs by allowing them to avoid their uptake by macrophages [14,15]. This prolonged in vivo residency time supports preferential localization in tumor environments through their leaky tumor vasculature [16]. However, studies have shown that having PEG on NPs could reduce their uptake once they reach tumor cells. Therefore, one of the approaches used to boost NP uptake is to add a peptide containing arginine-glycine-aspartic acid (RGD) sequence to NPs in addition to PEG [16,17]. The design of multifunctional nanocarriers to improve current therapeutic applications also requires a thorough understanding of their delivery in vivo followed by their uptake by individual cells [18].

### 3.1. GNPs as Radiosensitizers

Delivering doses that would eradicate tumor tissue without disrupting surrounding healthy tissues has been a challenge and it is still being studied to a great extent to reach the optimal and delicate balance. Targeting tumor cells using high-Z materials has been pursued to improve the local radiation dose and thus minimize the damage to surrounding healthy tissue [19]. The interaction between radiation (therapeutic X-ray photons) and high-Z materials within tumor tissue results in an increase in the local cross-section of cell damaging species, such as free radicals and low energy electrons [20,21]. This phenomenon opened the door for NP systems incorporating high-Z elements to be investigated as radiosensitizers [22,23]. NPs are particles between 1–1000 nanometers and often have different properties than those seen in bulk material of the same composition. NPs can be tailored for specific needs by modifying their size, shape, and surface properties [24,25,26]. Inorganic NP systems such as GNPs, silver NPs, gadolinium-based NPs, lanthanide-based NPs, and titanium oxide nanotubes have been reported as radiosensitizers [27,28,29,30,31,32,33]. Gadolinium-based NPs offer an innovative approach because of their capacity to act as a radiosensitizer as well as a powerful contrast agent in magnetic resonance imaging [32]. The high-Z nature of silver-based NPs along with their antimicrobial properties made them a good candidate in RT [33]. GNPs particularly are the most promising due to their simple surface chemistry, biocompatibility, and ease of manufacture [24,31,34,35,36,37].

**Figure 3:**
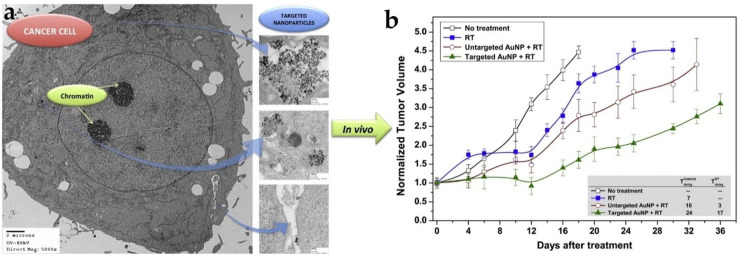
Gold nanoparticle (GNP)-mediated radiotherapy (RT). (**a**) TEM image of a prostate cancer with internalized gold nanorods. (**b**) In vivo radiosensitization of gold nanorod-incorporated tumor with megavoltage X-rays results in a substantial increase in tumor growth delay. This foretells the possibility of clinical translational of this nanomedicine approach to enhance RT. Reproduced with permission from [18], Elsevier, 2015.

One of the pioneering studies by Hainfeld et al. demonstrated GNP-mediated radiosensitization in a mice tumor graft where 1.9 nm GNPs and 30 Gy of 250 kilovoltage-peak (kVp) X-rays was used. GNPs plus radiation resulted in 86% long-term survival compared to 20% with radiation alone [36]. GNPs are radiation dose enhancers and the dose enhancement is attributed to the production of secondary electrons scattering from the surface of the high-Z material as discussed next [38,39]. The kV energies used in this study can be used treat superficial tumors due to the lack of penetration power of these low radiation energies. In the clinic, the deep seated tumors are treated with higher energy photons with mega-voltage energy range from 4 MV to 25 MV [40]. Both in vitro and in vivo studies have demonstrated the promise of GNPs with megavoltage radiation to generate radiosensitization [18,31,41]. For example, Krishnan and co-workers used goserelin-conjugated gold nanorods in prostate cancer to demonstrate the associated sensitization in vivo (see Figure 3) [18]. When systemically administered, conjugated gold nanorods accumulated preferentially in prostate cancers in vivo, and subsequent irradiation with megavoltage X-rays resulted in a substantial increase in tumor growth delay. Most importantly, there was a 3-fold higher radiosensitization to MV radiation with targeted NPs compared untargeted ones (Figure 3b). This suggests that radiosensitization is improved by active targeting of NPs which led to improved cellular internalization of the NPs. The presence of NPs in cells resulted in an increase in ionization density within the cytoplasm, rather than merely a passive accumulation of NPs in the perivascular space by the enhanced permeability and retention (EPR) effect [18]. Their results demonstrate that megavoltage radiosensitization was achievable in vivo using tumor-targeting NPs administered intravenously at a low concentration of 10 mg/kg body weight of gold. This is over one thousand-fold lower than that previously thought to be necessary for radiosensitization [36]. The use of megavoltage radiation to generate such radiosensitization in vivo at such clinically feasible concentrations of NPs foretell the possibility of eventual clinical translation of this approach.

#### Radiosensitization Mechanisms of GNPs

**Figure 4:**
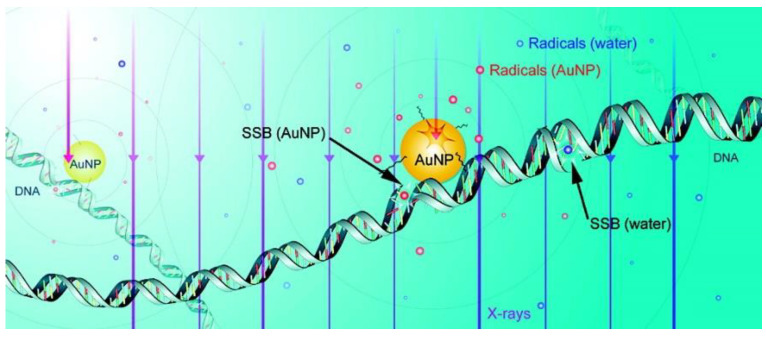
Schematic showing chemical mechanism of GNP radiosensitization. Reproduced with permission from [42], American Chemical Society, 2017.

This radiosensitization effect is thought to result from an increased number of photoelectric absorption events and the increased number of electrons present in gold. This increase in the number of electrons triggers an increase in free radicals which leads to a rise in DNA damage upon irradiation and therefore amplifies cell death numbers (see Figure 4) [42,43]. These species can do damage directly to the DNA or to other biomolecules or cause oxidative stress which can lead to apoptosis or necrosis (unregulated cell death due to stress) [44]. However, the exact mechanism is not fully known yet. While the relative dose enhancement and range of these electrons is higher when using a kilovoltage X-ray beam, meaningful enhancement can still be achieved with megavoltage beams due to contamination of the beam by lower-energy scattered photons and electrons produced as it passes through tissue [45]. For example, a dose enhancement factor of 1.17 for 6 MV radiation vs. 1.66 at 105 kVp [31]. This means that deep-seated tumors that cannot be effectively treated with shallowly-penetrating kilovoltage beams could still benefit from GNP-enhanced RT as illustrated in the previous section.

## 4. Prospects of Using GNPs with Other Radiosensitizers

The integration of RT with other modalities, such as chemotherapy, is a logical and reasonable approach that has greatly improved the cure rates of solid tumors [46,47]. Clinically, a combined approach of RT and chemotherapy is used for local control of the primary tumor mass through radiation and control of the metastatic disease through systemic chemotherapy [47]. For cancer treatment, the possible choice of chemotherapeutic drugs with RT could vary depending on the tumor type and the stage of the cancer. Among the variety of clinically available chemotherapeutic drugs, drugs such as cisplatin and DTX show the best promise as radiosensitizing agents. The use of GNPs in combination with current clinically available radiosensitizing agents will provide an optimum approach to control the disease both locally and systemically.

### 4.1. GNPs with Cisplatin

This section will discuss the use of cisplatin as a radiosensitizing agent followed by its use in combination treatment with GNPs.

#### 4.1.1. Cisplatin as a Radiosensitizer

**Figure 5:**
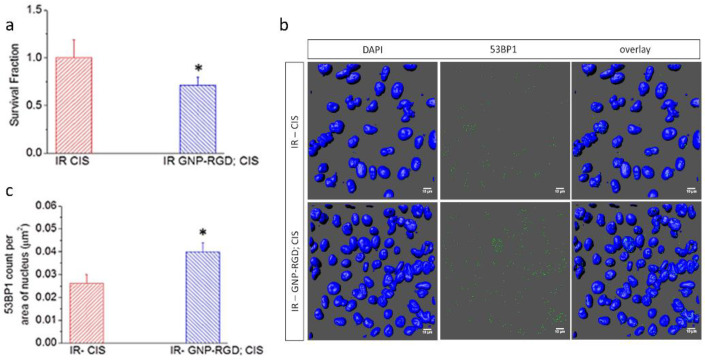
Combination with GNPs and cisplatin in RT. (**a**) Cell survival fraction of MDA-MB-231 cells treated with cisplatin (CIS) and GNP- arginine-glycine-aspartic acid (RGD); CIS prior to 2 Gy, 6 MV radiation. Data are means ± S.E.M. for *n* = 3. * represents statistically significant difference (unpaired *t*-test, *p* < 0.05); (**b**) Qualitative representation of DNA double-strand breaks (DSBs) in MDA-MB-231 cells treated with CIS and GNP-RGD; CIS prior to 2 GY, 6 MV X-ray radiation. The nucleus is stained with DAPI (shown in blue) and the markers for DNA DSBs, 53BP1, are shown in green. Scale bar = 10 µm; (**c**) Quantitative analysis of (**b**). Reproduced from [68], MDPI, 2018.

Another successful anticancer drug that is widely used today in conjugation with RT is *cis*-diamminedichloroplatinum(II), also known as cisplatin [48]. The anti-tumor properties of cisplatin were established in 1970, and the drug was approved by the FDA in 1978 [49,50,51,52]. This chemotherapeutic drug is used to treat ovarian, cervical, head and neck, esophageal, non-small cell lung, and testicular cancers [53,54,55,56,57]. The uptake pathway of this drug is known to be through passive diffusion [58]. Eljack et al. showed that cisplatin is capable of passive diffusion across the lipid bilayer membrane [59]. The structure of cisplatin (cis-[PtCl_2_(NH_3_)_2_]) has no net charge and is stable in the blood stream and in the extracellular matrix [58,59]. Once the compound enters the cytoplasm, the chloride ions dissociate from the platinum ion allowing it to react with cellular targets [58,59]. It is generally accepted that the primary target for cisplatin is the DNA. It forms a cisplatin–DNA cross-link agent that acts on the N7 position of guanine and adenine bases, resulting in distortion of the DNA [48,60,61,62]. The formation of this cross-link structure destroys the helix stability of the DNA [48]. Since DNA replication and transcription are essential for cell division and protein production, the cisplatin binding to the DNA and distorting the DNA structure is cytotoxic [48]. Studies showed that cisplatin inhibits DNA transcription, where transcription refers to the process where mRNA is produced from a DNA template. This cellular process is critical in protein synthesis [63,64]. According to these studies, cisplatin-treated cells progressed through the S phase of the cell cycle, where DNA synthesis happens, and were arrested in the G2 phase. For cells treated with a lower concentration of cisplatin, the G2 arrest was temporary. However, cells treated with higher concentrations remained in the G2 arrest until cell death occurred [63]. The mechanism of cell death from cisplatin was found to be through apoptosis [65]. It is a one of the common drugs used with radiation and has been previously tested with GNPs and with MDA-MB-231 cells [66]. That makes it an ideal drug to use in a combined cancer therapy study (see Figure 5). However, the significant risk of nephrotoxicity frequently hinders the use of higher doses to maximize its antineoplastic effects [67]. Therefore, novel therapeutic interventions aimed at minimizing cisplatin-induced nephrotoxicity while enhancing its antineoplastic efficacy is needed. For example, an efficient delivery of the drug to the tumor using a NP formulation or use of a combination of cisplatin with another radiosensitizing agents may allow a feasible therapeutic approach to increase the RT dose.

#### 4.1.2. Combination of Cisplatin and GNPs in RT

The combination of cisplatin and GNPs with radiation resulted in an additive radiosensitization effect both in vitro and in vivo [68,69]. As illustrated in Figure 5, the outcome of GNP-mediated chemoradiation was assessed by measuring the cell survival fraction and DNA damage in vitro [68]. DNA double-strand breaks (DSBs) are considered the most harmful type of DNA lesions because unrepaired DSBs are sufficient to trigger permanent growth arrest and cell death. More importantly, significant radiation enhancement was achieved by the combination of GNPs and cisplatin in vivo as well [69]. Based on tumor growth suppression and overall survival, it is suggested that the combination of GNP with cisplatin is a potent strategy to improve the outcome of RT by allowing additive therapeutic results corresponding to both studies. In contrast, synergistic therapeutic results were reported in a plasmid DNA model where GNPs and cisplatin were in direct contact with DNA during RT [37]. Therefore, it is theorized that further synergistic therapeutic improvements could be achieved if GNPs could directly target the nucleus in the presence of cisplatin and ionizing radiation [70,71].

**Figure 6:**
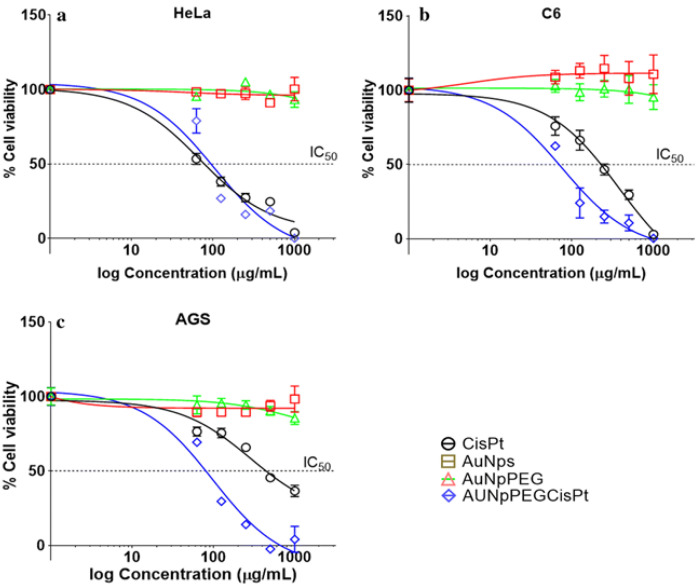
Use of GNPs as drug delivery systems for cisplatin. Cell viability curve of (**a**) HeLa cells, (**b**) C6 cells, and (**c**) AGS cells. Reproduced with permission from [72], American Chemical Society, 2007.

Furthermore, GNPs could be used as a cisplatin delivery vehicle [72]. A recent study showed that if GNPs are used as drug carriers, the amount of cisplatin necessary to cause the desired cytotoxic effect would be less than the amount used in conventional cancer treatments [72]. For example, IC_50_ diminished approximately 7 times, compared to the IC_50_ of cisplatin used alone, when using GNPs as delivery vehicles (see Figure 6) [72]. On this note, a new methodology has been developed for the synthesis of a gold core–shell type NP. It contains cisplatin within the shell which may facilitated its transport across the cell membrane. Therefore, this study opens up the possibility of an alternative way of transporting cisplatin anticancer drug to tumors using GNPs. This approach reduces the required dose but would have the same therapeutic effects as free cisplatin while minimizing undesired side effects. Another approach tested for GNP-mediated drug delivery is to conjugate cisplatin to the NP surface via a pH sensitive bond [73]. Local release of cisplatin from the formulation was successfully accomplished by exploiting the acidic tumor microenvironment and thereafter achieving a tumor-specific delivery of cisplatin [74]. Therefore, engineering such GNP-based formulations will improve the therapeutic window for using cisplatin and GNPs in RT.

### 4.2. GNPs with Docetaxel

#### 4.2.1. Docetaxel as a Radiosensitizer

DTX is a semi-synthetic drug derived from taxanes, originally identified in the Yew tree (genus *Taxus*). It is a commonly used chemotherapy drug for the treatment of a number of cancers including breast, stomach, prostate, and non-small-cell lung cancer [75,76]. DTX was patented in 1986 and approved for medical use in 1995. DTX acts as a radiosensitizer by blocking cells in G2 and mitosis phases, the most radiosensitive phases of the cell cycle. DTX alone has shown remarkable radiosensitization both in vitro and in vivo [77,78,79,80]. It has also been investigated as a radiosensitizer in several Phase II clinical trials [81,82,83,84,85].

**Figure 7:**
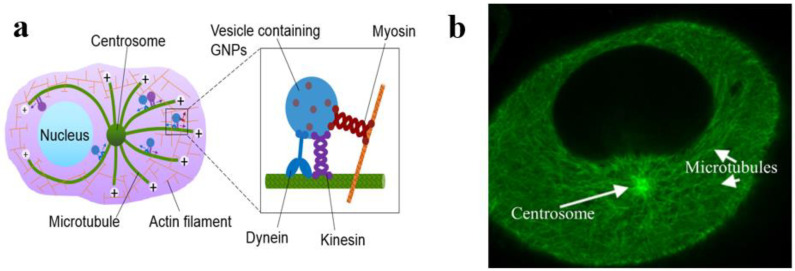
Microtubule network within a cell. (**a**) A diagram showing the transport of vesicles containing GNPs within the microtubule network. (**b**) A snapshot of a live cell showing a high number of microtubules throughout the cell. Reproduced from [86], MDPI, 2020.

In order to explain the mechanism action of DTX, it is important to understand the role of microtubules (MTs) within a cell. MTs are a major component of the cytoskeleton of the cell that facilitate cell morphology and motility. They also provide a framework for the localization of organelles and transport of cargoes within the cell as illustrated in Figure 7. MTs are composed of α- and β-tubulin proteins, which form dimers that float free in the cytoplasm. MTs are nucleated in the microtubule organizing center (MTOC) near the nucleus of the cell (Figure 7b)**.** Tubulin dimers are recruited to link in end-to-end chains and then “zip” together to form hollow MTs. They are polarized, with a negatively charged end (−) at the nucleation site and polymerization occurring at the (+) end alternately growing or shrinking depending on its local environment. Many cargoes within the cell are transported throughout the cell by motor proteins along these MTs. They also play a critical role in cell division via composing the mitotic spindle that allows for DNA segregation into daughter cells.

**Figure 8:**
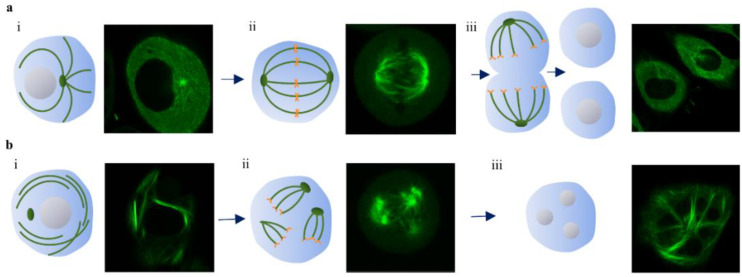
Schematic diagram and confocal images of cell division showing microtubules under normal conditions (**a**) and 50 nM DTX (**b**). (**a**) (i) Quiescent normal cell, (ii) normal mitotic spindle, (iii) normal pair of daughter cells. (**b**) (i) Asters formation in non-dividing cell with DTX, (ii) fragmentary division with 10 nM DTX, and (iii) mitotic arrest with multinucleation under 50 nM DTX. Adapted with permission from [92], Europe PMC, 1997.

The action of the drug DTX is through binding to the β-tubulin of MT. This results in stabilizing it against depolymerization, while also lowering the critical concentration of tubulin necessary to form new MTs [87]. This would lead to unregulated formation of MTs, without regard to the MTOC, resulting in bundles hindering the formation of a mitotic spindle which is necessary for cell division (Figure 8a) [88,89,90,91,92]. With DTX treatment, asters and bundles are formed independently of centrosomes, creating multiple cleavage planes. For example, 50 nM DTX is sufficient to cause a “mitotic catastrophe”: the cell cannot enter anaphase and remains locked in mitosis or becomes multinucleate as the nuclear envelope reforms around the multiple asters (Figure 8b). This results in blocking the cell cycle at the G2/M phase and it is the most sensitive phase to radiation. This is why DTX act as a radiosensitizer in RT.

#### 4.2.2. Combination of DTX and GNPs in RT

DTX is being used with RT to treat locally advanced prostate cancer patients. Phase III clinical trials show positive results [91]. A recent study shows that the addition of GNPs into current DTX/RT would produce a very promising synergistic therapeutic result [92]. This smart combination of cancer therapy is proposed to reduce the required dosages, thus minimizing the damage to healthy tissue surrounding the tumor [93]. GNPs act as a radiosensitizer by boosting local ionizing radiation doses to cancer cells as discussed before. Gold has a large cross-section for lower energy photons, consequently they absorb the energy of the incident photons and eject electrons in the vicinity of a cancer cell nucleus damaging the DNA and resulting in cell death. On the other hand, DTX has shown remarkable effects when used alone as a cancer therapeutic drug or in combination with ionizing radiation when used as a radiosensitizer [92]. When in use with ionizing radiation, DTX shows radiosensitization effects by blocking cells at the most radiosensitive phases of the cell cycle, i.e., the G2/M phases. The synergistic effect of the triple combination of GNPs, DTX, and ionizing radiation has a massive prospect to revolutionize cancer treatment.

**Figure 9:**
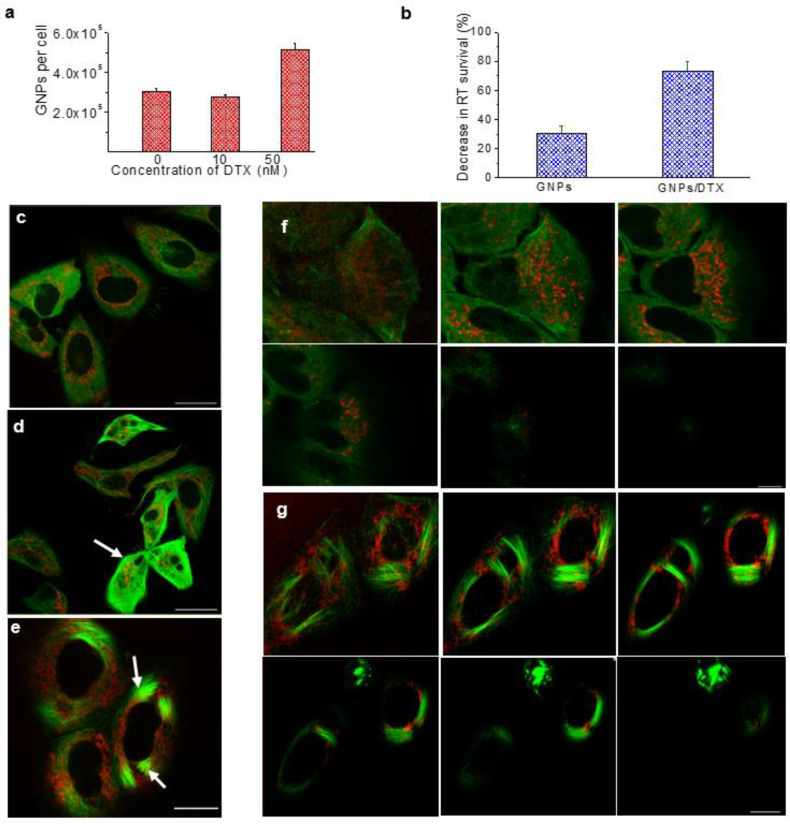
Triple combination of RT/DTX/GNPs. (**a**) GNP uptake as a function of DTX concentration in HeLa cells (24 h exposure). (**b**) Comparison of percent decrease in survival fraction in RT with the addition of GNPs vs. GNPs/DTX in HeLa cells (Adapted from Bannister et al. [39]). (**c**–**e**) Confocal images of GNPs (in red) and microtubules (in green) in HeLa cell with the exposure 0, 10, and 50 nM of DTX, respectively. (**f**,**g**) The distribution of GNPs across few planes of HeLa cells treated with 0 and 50 nM of DTX, respectively. The scale bar is 25 µm. Reproduced from [86], MDPI, 2020.

Figure 9 shows a comparison of the triple combination of RT/GNPs/DTX vs. RT/DTX without GNPs in a HeLa cell line [92]. The GNPs’ uptake increases almost 70% in the presence of DTX after 24 h of incubation (Figure 9a). The largest number of GNP accumulation is in the G2/M phase due to DTX halting cell division at this phase and allowing more time for GNPs to be accumulated before division. Figure 9b demonstrates the effect of the addition of GNPs into the RT/DTX treatment. It is very evident that the addition of GNPs considerably decreased the survival fraction of HeLa cells which indicates the efficacy of the two radiosensitizers’, GNPs/DTX, synergic effect. It has also been noted that cells treated with DTX changed their morphology due to bundling of microtubules (Figure 9c,e). Distribution of NPs in control cells and ones treated with DTX across few planes is illustrated in Figure 9f,g, respectively. In order to make this approach safer and more effective, GNP-based nanoformulations could be used as promising vehicles for a controlled and efficient delivery DTX vs. using free DTX. The tunable size and shape, biocompatibility of GNPs, and easiness of conjugation to biomolecules allow making such formulations feasible.

**Figure 10:**
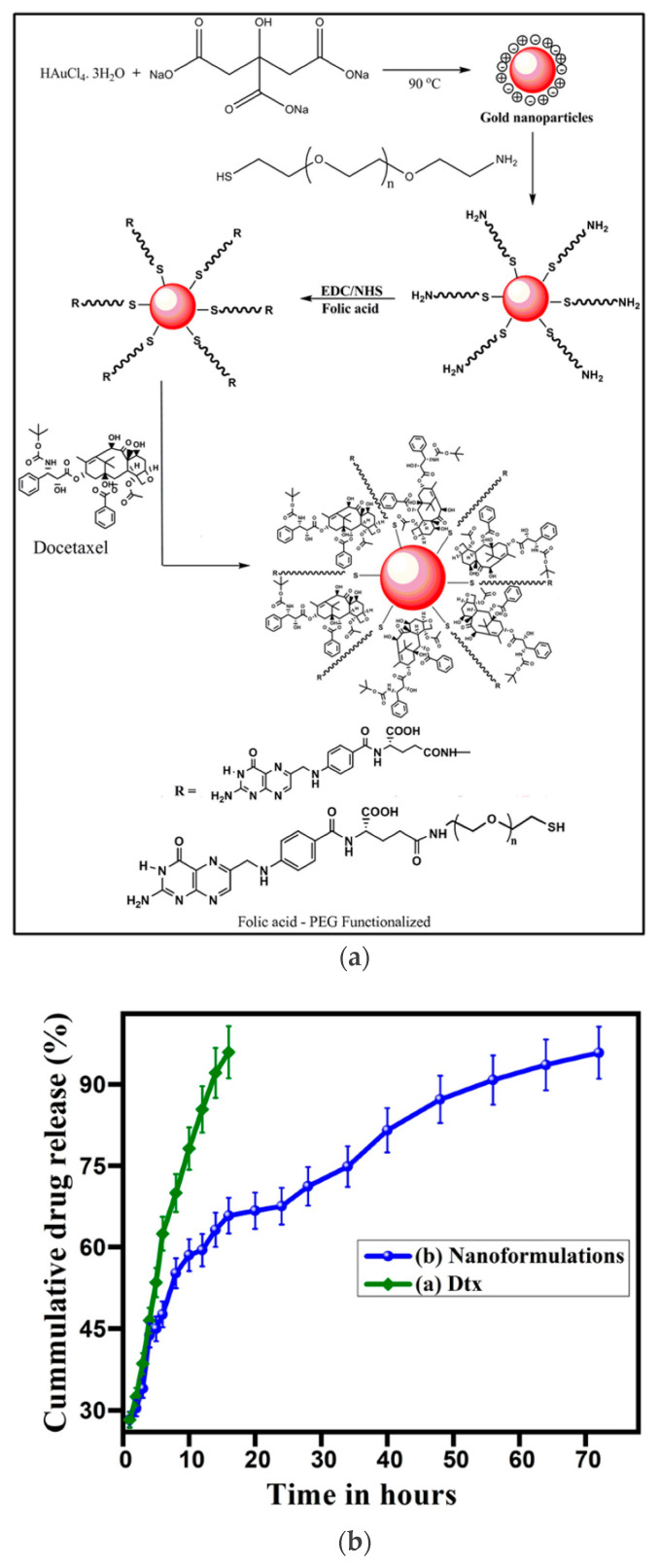
Use of GNPs for delivery of DTX. (**a**) Mechanism for the synthesis of DTX incorporated GNPs. (**b**) In vitro drug release profile of (**a**) DTX and (**b**) gold nano-formulations in PBS solution at pH 7.4. Reproduced from [94], Springer Nature, 2021.

A recent study has established a simple method for the synthesis of DTX encapsulated polyethylene glycol (PEG) functionalized GNPs (see Figure 10a) for targeted drug delivery to prostate cancer [94]. The encapsulation efficiency of DTX was ~96%. In vitro drug release kinetics of optimized DTX-encapsulated GNP nano-formulations and the free drug was investigated by the dialysis membrane method. The results are shown in Figure 10b. The anticancer activity of DTX-encapsulated GNPs were evaluated with prostate cancer cell lines (PC3) and the drug encapsulated GNPs reduced the cell viability to about 40%. The good cytotoxic activity of DTX-conjugated GNPs against cancer cell lines enables its application for targeted drug delivery to prostate cancer.

## 5. Future Prospects of Radiotherapy with Other Radiosensitizing Agents

Currently, RT is the most employed therapeutic modality for the treatment of cancer, in addition to chemotherapy and surgery. The main issue in RT is the normal tissue toxicity.The pressing need to overcome challenges in current cancer therapeutic options led to the development of the interdisciplinary field of cancer nanomedicine and the use of nanoscale materials as novel assisting treatment tools [95]. One of the current approaches to manage this hurdle is to add radiosensitizing agents to the tumor region to enhance the local radiation dose. GNP systems, while they have shown remarkable radiosensitizing properties in vitro and in vivo, still require refinement and optimization before reaching widespread clinical use [24]. At this time, there are only two GNP systems in clinical trials for cancer: one using tumor necrosis factor-conjugated GNPs (NCT00356980 and NCT00436410 from www.clinicaltrials.gov, accessed on 24 March 2021), and one using gold nanorods for photothermal ablative therapy under the name Aurolase (NCT00848042 and NCT02680535 from www.clinicaltrials.gov, accessed on 24 March 2021). Neither is specified for radiosensitization, nor has yet made it to stage II trials. However, GNP-induced radiosensitization has been demonstrated in several cell lines and in murine models, yet the sheer variety of sizes, shapes, and surface coating combinations being used make it difficult to determine what the optimal parameters would be for a clinical formulation [24,96]. The combination of GNPs with other clinically approved radiosensitizing agents to improve current RT should be considered. GNPs have huge potential as targeted vectors for the delivery of other radiosensitizing agents to create a much more desirable approach to treat cancer. DTX and cisplatin stand out as potential chemotherapeutic drugs that could be utilized as prospective radiosensitizing agents with GNPs and RT in a treatment system that would deliver effective synergic damage to tumors.

## 6. Conclusions

We have unveiled a nanomedicine-based platform to further enhance the current RT dose. The goal of this article was to unveil what has been achieved so far and what is left to be done to combine GNPs with other clinically available radiosensitizing drugs. We reviewed docetaxel and cisplatin since they are widely used radiosensitizing drugs in the clinic. The combination of these available radiosensitizing drugs with GNPs would allow increasing the efficacy of the treatment while minimizing normal tissue toxicity. Nanomedicine will revolutionize future cancer treatment options and the ultimate goal should be to develop treatments that have limited or non-existent side effects to improve the quality of life of all cancer patients. This would require a much closer collaboration among radiation oncologists, medical oncologists, and other scientists to cross barriers in introducing this latest technology to the clinic in the near future.

## Data Availability

Not applicable.
